# Supplementary material for: Antagonism between parasites within snail hosts impacts the transmission of human schistosomiasis
Source: eLife. 2019 Dec 17;8:e50095. doi: 10.7554/eLife.50095 (PMC6917487; doi:10.7554/eLife.50095)
Supplement: Supplementary file 1. [file elife-50095-supp1.pdf]

Supplementary Table 1: Description of Model Variables

Variable	Description
M_{Sm}	*S. mansoni* miracidia
M_{Cs}	*C. sukari* miracidia
S_{ss}	Susceptible sink snails
I_{ss}	Infected sink snails
S_j	Susceptible juvenile *Biomphalaria*
S_a	Susceptible adult *Biomphalaria*
E_{jSm}	Exposed juvenile *Biomphalaria* - *S. mansoni* only
E_{aSm}	Exposed adult Biomphalaria - *S. mansoni* only
E_{jCs}	Exposed juvenile *Biomphalaria* - *C. sukari* only
E_{aCs}	Exposed adult *Biomphalaria* - *C. sukari* only
E_{jSi}	Exposed juvenile *Biomphalaria* - Simultaneous infection
E_{aSi}	Exposed adult *Biomphalaria* - Simultaneous infection
E_{jSmCs}	Exposed juvenile Biomphalaria - *S. mansoni* then *C. sukari*
E_{aSmCs}	Exposed adult *Biomphalaria* - *S. mansoni* then *C. sukari*
E_{jCsSm}	Exposed juvenile *Biomphalaria* - *C. sukari* then *S. mansoni*
E_{aCsSm}	Exposed adult *Biomphalaria* - *C. sukari* then *S. mansoni*
I_{jSm}	Infected juvenile *Biomphalaria* - *S. mansoni* only
I_{aSm}	Infected adult Biomphalaria - *S. mansoni* only
I_{jCs}	Infected juvenile *Biomphalaria* - *C. sukari* only
I_{aCs}	Infected adult *Biomphalaria* - *C. sukari* only
I_{jSi}	Infected juvenile *Biomphalaria* - Simultaneous infection
I_{aSi}	Infected adult *Biomphalaria* - Simultaneous infection
I_{jSmCs}	Infected juvenile Biomphalaria - *S. mansoni* then *C. sukari*
I_{aSmCs}	Infected adult *Biomphalaria* - *S. mansoni* then *C. sukari*
I_{jCsSm}	Infected juvenile *Biomphalaria* - *C. sukari* then *S. mansoni*
I_{aCsSm}	Infected adult *Biomphalaria* - *C. sukari* then *S. mansoni*
I_{cSm}	Infected & castrated adult *Biomphalaria* - *S. mansoni* only
I_{cCs}	Infected & castrated adult *Biomphalaria* - *C. sukari* only
I_{cSi}	Infected & castrated adult *Biomphalaria* - Simultaneous infection
I_{cSmCs}	Infected & castrated adult *Biomphalaria* - *S. mansoni* then *C. sukari*
I_{cCsSm}	Infected & castrated adult *Biomphalaria* - *C. sukari* then *S. mansoni*
N_B	Sum of all *Biomphalaria* snail variables (Susceptible, Exposed and Infected)
C_{Sm}	*S. mansoni* cercariae
C_{Cs}	*C. sukari* cercariae
