# Supplementary material for: Antagonism between parasites within snail hosts impacts the transmission of human schistosomiasis
Source: eLife. 2019 Dec 17;8:e50095. doi: 10.7554/eLife.50095 (PMC6917487; doi:10.7554/eLife.50095)
Supplement: Supplementary file 2. [file elife-50095-supp2.pdf]

## Supplementary Table 2: Description of Parameter Values

| Parameter | Description | Value(s) | References |
|---|---|---|---|
| $g_1$ | Input of *S. mansoni* miracidia | 500-10000 /week | (a) |
| $g_2$ | Input of *C. sukari* miracidia | 150000-250000 /week | (a) |
| $e_1$ | Exposure rate of sink snails to *S. mansoni* miracidia | 0.06/week | (a) |
| $e_2$ | Exposure rate of juvenile *Biomphalaria* snails to *S. mansoni* miracidia | 0.18/week | (a) |
| $e_3$ | Exposure rate of adult *Biomphalaria* snails to *S. mansoni* miracidia | 0.18/week | (a) |
| $e_4$ | Exposure rate of sink snails to *C. sukari* miracidia | 0.06/week | (a) |
| $e_5$ | Exposure rate of juvenile *Biomphalaria* snails to *C. sukari* miracidia | 0.18/week | (a) |
| $e_6$ | Exposure rate of adult *Biomphalaria* snails to *C. sukari* miracidia | 0.18/week | (a) |
| $e_7$ | Exposure rate of juvenile *Biomphalaria* snails to simultaneous infection | 0.0018/week | (a) |
| $e_8$ | Exposure rate of adult *Biomphalaria* snails to simultaneous infection | 0.0018/week | (a) |
| $1/y_1$ | Prepatent period for *S. mansoni* infection | 4.5 weeks | (1) |
| $1/y_2$ | Prepatent period for *C. sukari* infection | 4 weeks | (b) |
| $1/c_1$ | Time until *Biomphalaria* castration with *S. mansoni* only infection | 6 weeks | (2) |
| $1/c_2$ | Time until *Biomphalaria* castration with *C. sukari* only infection | 4 weeks | (b) |
| $1/c_3$ | Time until *Biomphalaria* castration with dual infection | 4 weeks | (b) |
| $f_1$ | Fecundity per susceptible sink snail | 14 eggs/week | (2,3,4,5,6) |
| $f_2$ | Fecundity per susceptible *Biomphalaria* snail | 20-3 eggs/week | (8,3,7,2,5) |
| $f_3$ | Fecundity per adult *Biomphalaria* exposed or infected with *S. mansoni* or *C. sukari* | 4.77 eggs/week | (3,4,7,2,5) |
| $1/a_1$ | Duration of juvenile stage for susceptible *Biomphalaria* | 7 weeks | (9,10,11) |
| $1/a_2$ | Duration of juvenile stage for *Biomphalaria* exposed or infected with *S. mansoni* | 9.8 weeks | (9,10,11) |
| $1/a_3$ | Duration of juvenile stage for *Biomphalaria* exposed or infected with *C. sukari* | 9.8-15 weeks | (9,10,11) |
| $1/a_4$ | Duration of juvenile stage for simultaneously infected *Biomphalaria* | 9.8-15 weeks | (9,10,11) |
| $1/d_1$ | Lifespan of *S. mansoni* or *C. sukari* miracidia | 4-16 hours | (9,12) |
| $d_2$ | Mortality rate of susceptible snails | 1/18.2 /week | (9,12) |
| $d_3$ | Mortality rate of exposed or infected snails | 1/8.1 /week | (8) |
| $1/d_4$ | Lifespan of *S. mansoni* or *C. sukari* cercariae | 8-20 hours | (13) |
| $w$ | Liters of water in the environment | 4180 | (c) |
| $m_1$ | Maximum number of *Biomphalaria* in the environment | 2000 | (c) |
| $m_2$ | Maximum number of sink snails in the environment | 500 | c |

| Parameter | Description | Value(s) | References |
|---|---|---|---|
| $u_1$ | Rate of uptake of *S. mansoni* cercariae by humans | .5% /week | a |
| $u_2$ | Rate of uptake of *C. sukari* cercariae by humans | .5% /week | a |
| $s_1$ | Rate of *S. mansoni* cercarial shedding for $I_{jSm}$ | 4125/week | (7,10) |
| $s_2$ | Rate of *S. mansoni* cercarial shedding for $I_{aSm}$ | 5100/week | (b) |
| $s_3$ | Rate of *S. mansoni* cercarial shedding for $I_{jSi}$ | 14/week | (b) |
| $s_4$ | Rate of *S. mansoni* cercarial shedding for $I_{jSmCs}$ | 10/week | (b) |
| $s_5$ | Rate of *S. mansoni* cercarial shedding for $I_{aSmCs}$ | 10/week | (b) |
| $s_6$ | Rate of *S. mansoni* cercarial shedding for $I_{jCsSm}$ | 7/week | (b) |
| $s_7$ | Rate of *S. mansoni* cercarial shedding for $I_{aCsSm}$ | 7/week | (b) |
| $s_8$ | Rate of *S. mansoni* cercarial shedding for $I_{aSi}$ | 5100/week | (b) |
| $s_9$ | Rate of *S. mansoni* cercarial shedding for $I_{cSm}$ | 140/week | (b) |
| $s_{10}$ | Rate of *S. mansoni* cercarial shedding for $I_{cSi}$ | 140/week | (b) |
| $s_{11}$ | Rate of *S. mansoni* cercarial shedding for $I_{cSmCs}$ | 14/week | (b) |
| $s_{12}$ | Rate of *S. mansoni* cercarial shedding for $I_{cCsSm}$ | 10/week | (b) |
| $s_{13}$ | Rate of *C. sukari* cercarial shedding for $I_{jCs}$ | 10/week | (b) |
| $s_{14}$ | Rate of *C. sukari* cercarial shedding for $I_{aCs}$ | 5.6/week | (b) |
| $s_{15}$ | Rate of *C. sukari* cercarial shedding for $I_{jSi}$ | 5.6/week | (b) |
| $s_{16}$ | Rate of *C. sukari* cercarial shedding for $I_{jSmCs}$ | 140/week | (b) |
| $s_{17}$ | Rate of *C. sukari* cercarial shedding for $I_{aSmCs}$ | 140/week | (b) |
| $s_{18}$ | Rate of *C. sukari* cercarial shedding for $I_{jCsSm}$ | 280/week | (b) |
| $s_{19}$ | Rate of *C. sukari* cercarial shedding for $I_{aCsSm}$ | 280/week | (b) |
| $s_{20}$ | Rate of *C. sukari* cercarial shedding for $I_{aSi}$ | 140/week | (b) |
| $s_{21}$ | Rate of *C. sukari* cercarial shedding for $I_{cCs}$ | 140/week | (b) |
| $s_{22}$ | Rate of *C. sukari* cercarial shedding for $I_{cSi}$ | 140/week | (b) |
| $s_{23}$ | Rate of *C. sukari* cercarial shedding for $I_{cSmCs}$ | 140/week | (b) |
| $s_{24}$ | Rate of *C. sukari* cercarial shedding for $I_{cCsSm}$ | 140/week | (b) |

(a) Parameters calibrated from prevalence data; (b) Parameters estimated directly from laboratory experiments; (c) Parameters estimated directly from field data.


1. Cooper, L. A., Ramani, S. K., Martin, A. E., Richards, C. S., Fred, A., Cooper, L. A Lewis, F. A. (1992). Schistosoma mansoni Infections in Neonatal Biomphalaria glabrata Snails. Journal of Parasitology, 78(3), 441 – 446.

2. Crews, A. E., Yoshino, T. P. (1989). Schistosoma mansoni: Effect of Infection on Reproduction and Gonadal Growth in Biomphalaria glabrata. Experimental Parasitology, 68, 326 – 334.

3. Costa, M. F. F. D. L., Rocha, R. S., Filho, P. C., Katz, N. (1993). A 13-year follow-up of treatment

and snail control in an area endemic for Schistosoma mansoni in Brazil?: incidence of infection and reinfection. Bulletin of the World Health Organization, 71(2), 197 – 205.

4. Anderson, R. M., Crombie, J. (1984). Experimental studies of age-prevalence curves for Schistosoma mansoni infections in populations of Biomphalaria glabrata. Parasitology, 89, 79 –104.

5. Costa, M. J. F. S., Grault, C. E., Confalonieri, U. E. C. (2004). COMPARATIVE STUDY OF THE FECUNDITY AND FERTILITY OF Biomphalaria glabrata ( Say , 1818 ) AND Biomphalaria straminea ( Dunker , 1848 ) IN A LABORATORY THROUGH SELF-FERTILIZATION AND CROSS-FERTILIZATION. Revista Do Instituto de Medicina Tropical de So Paulo, 46(3), 157 –163.

6. Woolhouse, M. E. (1989). The effect of schistosome infection on the mortality rates of Bulinus globosus and Biomphalaria pfeifferi. Annals of Tropical Medicine and Parasitology, 83(2), 137–141.

7. Gerard, C., Theron, A. (1997). Age/Size and Time Specific Effects of Schistosoma mansoni on Energy Allocation Patterns of Its Sail Host Biomphalaria glabrata. Oecologia, 112(4), 447– 452.

8. Theron, A., Rognon, A., Pages, J.-R. (1998). Host choice by larval parasites?: a study of Biomphalaria glabrata snails and Schistosoma mansoni miracidia related to host size. Parasitology Research, 84, 727–732.

9. Toledo R, Fried B. 2011. Biomphalaria Snails and Larval Trematodes. Springer-Verlag.

10. Anderson R, May RM. 1991. Infectious diseases of humans: dynamics and control. Oxford University Press

11. Faro, J. M., Perazzini, M., dos Reis Corra, L., Mello-Silva, C. C., Pinheiro, J., Mota, E. M., ? Maldonado, A. (2013). Biological , biochemical and histopathological features related to parasitic

castration of Biomphalaria glabrata infected by Schistosoma mansoni. Experimental Parasitology, 134(2), 228–234.

12. Niemann, G. M., Lewis, F. A. (1990). Schistosoma mansoni: Influence of Biomphalaria glabrata size on susceptibility to infection and resultant cercarial production. Experimental Parasitology, 70, 286–292.

13. Chiyaka, E. T., Garira, W. (2009). Mathematical analysis of the transmission dynamics of schistosomiasis in the human-snail hosts. Journal of Biological Systems, 17(3), 397–423.
