# Supplementary material for: Antagonism between parasites within snail hosts impacts the transmission of human schistosomiasis
Source: eLife. 2019 Dec 17;8:e50095. doi: 10.7554/eLife.50095 (PMC6917487; doi:10.7554/eLife.50095)
Supplement: Supplementary file 3. [file elife-50095-supp3.pdf]

Supplementary Information - Model Equations

$$
\begin{aligned}
\frac{dM_{Sm}}{dt} &= g_1 - \left(\frac{e_1(S_{ss}+I_{ss}) + e_2(S_j+E_{jCs}) + e_3(S_a+E_{aCs})}{w}\right)M_{Sm} \\
&\quad - \left(\frac{e_7 M_{Cs} S_j + e_8 M_{Cs} S_a}{w^2}\right)M_{Sm} - d_1 M_{Sm} \\
\frac{dM_{Cs}}{dt} &= g_2 - \left(\frac{e_4(S_{ss}+I_{ss}) + e_5(S_j+E_{jSm}) + e_6(S_a+E_{aSm})}{w}\right)M_{Cs} \\
&\quad - \left(\frac{e_7 M_{Sm} S_j + e_8 M_{Sm} S_a}{w^2}\right)M_{Cs} - d_1 M_{Cs} \\
\frac{dS_{ss}}{dt} &= (f_1 S_{ss} + f_3 I_{ss})\left(1 - \frac{S_{ss}+I_{ss}}{m_2}\right) - \left(\frac{e_1 M_{Sm} + e_4 M_{Cs}}{w}\right)S_{ss} - d_2 S_{ss} \\
\frac{dI_{ss}}{dt} &= \left(\frac{e_1 M_{Sm} + e_4 M_{Cs}}{w}\right)S_{ss} - d_3 I_{ss} \\
\frac{dS_j}{dt} &= (f_2 S_a + f_3(E_{aSm} + E_{aCs} + E_{aSi} + E_{aSmCs} + E_{aCsSm} + \\
&\quad I_{aSm} + I_{aCs} + I_{aSi} + I_{aSmCs} + I_{aCsSm}))\left(1 - \frac{N_B}{m_1}\right) \\
&\quad - \left(\frac{e_2 M_{Sm} + e_5 M_{Cs}}{w}\right)S_j - e_7\frac{M_{Sm}M_{Cs}}{w^2}S_j - d_2 S_j \\
\frac{dS_a}{dt} &= a_1 S_j - \left(\frac{e_3 M_{Sm} + e_6 M_{Cs}}{w}\right)S_a - e_8\frac{M_{Sm}M_{Cs}}{w^2}S_a - d_2 S_a \\
\frac{dE_{jSm}}{dt} &= e_2\frac{M_{Sm}}{w}S_j - e_5\frac{M_{Cs}}{w}E_{jSm} - (a_2 + y_1 + d_3)E_{jSm} \\
\frac{dE_{aSm}}{dt} &= a_2 E_{jSm} + e_3\frac{M_{Sm}}{w}S_a - e_6\frac{M_{Cs}}{w}E_{aSm} - (y_1 + d_3)E_{aSm} \\
\frac{dE_{jCs}}{dt} &= e_5\frac{M_{Cs}}{w}S_j - e_2\frac{M_{Sm}}{w}E_{jCs} - (a_3 + y_2 + d_3)E_{jCs}
\end{aligned}
$$

$$
\begin{aligned}
\frac{dE_{aCs}}{dt} &= a_3 E_{jCs} + e_6 \frac{M_{Cs}}{w} S_a - e_3 \frac{M_{Sm}}{w} E_{aCs} - (y_2 + d_3) E_{aCs} \\
\frac{dE_{jSi}}{dt} &= e_7 \frac{M_{Sm} M_{Cs}}{w^2} S_j - (a_4 + y_2 + d_3) E_{jSi} \\
\frac{dE_{aSi}}{dt} &= a_4 E_{jSi} + e_8 \frac{M_{Sm} M_{Cs}}{w^2} S_a - (y_2 + d_3) E_{aSi} \\
\frac{dE_{jSmCs}}{dt} &= e_5 \frac{M_{Cs}}{w} E_{jSm} - (a_2 + y_1 + d_3) E_{jSmCs} \\
\frac{dE_{aSmCs}}{dt} &= a_2 E_{jSmCs} + e_6 \frac{M_{Cs}}{w} E_{aSm} - (y_1 + d_3) E_{aSmCs} \\
\frac{dE_{jCsSm}}{dt} &= e_2 \frac{M_{Sm}}{w} E_{jCs} - (a_3 + y_2 + d_3) E_{jCsSm} \\
\frac{dE_{aCsSm}}{dt} &= a_3 E_{jCsSm} + e_3 \frac{M_{Sm}}{w} E_{aCs} - (y_2 + d_3) E_{aCsSm} \\
\frac{dI_{jSm}}{dt} &= y_1 E_{jSm} - (a_2 + d_3) I_{jSm} \\
\frac{dI_{aSm}}{dt} &= a_2 I_{jSm} + y_1 E_{aSm} - (c_1 + d_3) I_{aSm} \\
\frac{dI_{jCs}}{dt} &= y_2 E_{jCs} - (a_3 + d_3) I_{jCs} \\
\frac{dI_{aCs}}{dt} &= a_3 I_{jCs} + y_2 E_{aCs} - (c_2 + d_3) I_{aCs} \\
\frac{dI_{jSi}}{dt} &= y_2 E_{jSi} - (a_4 + d_3) I_{jSi} \\
\frac{dI_{aSi}}{dt} &= a_4 I_{jSi} + y_2 E_{aSi} - (c_3 + d_3) I_{aSi} \\
\frac{dI_{jSmCs}}{dt} &= y_1 E_{jSmCs} - (a_2 + d_3) I_{jSmCs} \\
\frac{dI_{aSmCs}}{dt} &= a_2 I_{jSmCs} + y_1 E_{aSmCs} - (c_3 + d_3) I_{aSmCs}
\end{aligned}
$$

$$
\begin{aligned}
\frac{dI_{jCsSm}}{dt} &= y_2 E_{jCsSm} - (a_3 + d_3) I_{jCsSm} \\
\frac{dI_{aCsSm}}{dt} &= a_3 I_{jCsSm} + y_2 E_{aCsSm} - (c_3 + d_3) I_{aCsSm} \\
\frac{dI_{cSm}}{dt} &= c_1 I_{aSm} - d_3 I_{cSm} \\
\frac{dI_{cCs}}{dt} &= c_2 I_{aCs} - d_3 I_{cCs} \\
\frac{dI_{cSi}}{dt} &= c_3 I_{aSi} - d_3 I_{cSi} \\
\frac{dI_{cSmCs}}{dt} &= c_3 I_{aSmCs} - d_3 I_{cSmCs} \\
\frac{dI_{cCsSm}}{dt} &= c_3 I_{aCsSm} - d_3 I_{cCsSm} \\
\frac{dC_{Sm}}{dt} &= s_1 I_{jSm} + s_2 I_{aSm} + s_3 I_{jSi} + s_4 I_{jSmCs} + s_5 I_{aSmCs} + s_6 I_{jCsSm} \\
&\quad + s_7 I_{aCsSm} + s_8 I_{aSi} + s_9 I_{cSm} + s_{10} I_{cSi} + s_{11} I_{cSmCs} + s_{12} I_{cCsSm} \\
&\quad - (u_1 + d_4) C_{Sm} \\
\frac{dC_{Cs}}{dt} &= s_{13} I_{jCs} + s_{14} I_{aCs} + s_{15} I_{jSi} + s_{16} I_{jSmCs} + s_{17} I_{aSmCs} + s_{18} I_{jCsSm} \\
&\quad + s_{19} I_{aCsSm} + s_{20} I_{aSi} + s_{21} I_{cCs} + s_{22} I_{cSi} + s_{23} I_{cSmCs} + s_{24} I_{cCsSm} \\
&\quad - (u_1 + d_4) C_{Cs}
\end{aligned}
$$
